# Central Tetrapolydactyly With Atrial Septal Defect and Facial Nerve Palsy in a 15-Month-Old Female Child

**DOI:** 10.7759/cureus.64915

**Published:** 2024-07-19

**Authors:** Janhavi V Thorat, Sampada Tambolkar, Shailaja Mane

**Affiliations:** 1 Paediatrics, Dr. D. Y. Patil Medical College, Hospital and Research Centre, Dr. D. Y. Patil Vidyapeeth (Deemed to be University), Pune, IND

**Keywords:** general pediatrics, congenital facial nerve palsy, genetic syndromes, congenital birth defect, pediatric genetics, post-axial polydactyly

## Abstract

Polydactyly, which is the presence of an extra appendage on the hand or the foot, is a common congenital anomaly encountered in children. It may be an isolated finding or found in conjunction with other congenital anomalies and syndromes. Polydactyly can occur in the hands or the feet. In the hand, it may occur as radial polydactyly (pre-axial polydactyly) or ulnar polydactyly (post-axial polydactyly (PAP)). Depending upon the side of occurrence, it may be medial, that is, toward the little finger (called ulnar polydactyly) or lateral, that is, toward the thumb (called radial polydactyly). On the feet, the extra digit can either be present on the side of the great toe (called tibial polydactyly) or on the side of the little toe (called fibular polydactyly). In both the upper and the lower limbs, affection of the central three digits is called central polydactyly. Central tetrapolydactyly, which is the presence of an extra appendage on all four limbs, is much more rarely encountered. This case report describes a 15-month-old female child who presented with findings of six digits on all four limbs and deviation of the left angle of mouth since birth. Her echocardiography showed a large atrial septal defect measuring 7 mm, with a left-to-right shunt. This is the first such case reported from all over the world from a tertiary care hospital with the aforementioned findings. Polydactyly, a very common congenital anomaly, should not be ignored in pediatric settings. It is important to diagnose associated features such as congenital heart diseases (CHDs), genitourinary abnormalities, and orofacial abnormalities to facilitate timely surgical correction and help improve the quality of life of those affected.

## Introduction

Polydactyly, which is the presence of an extra appendage on the hand or the foot, is a common congenital anomaly encountered in children. It may be an isolated finding or found in conjunction with other congenital anomalies and syndromes [1]. Comparatively, tetrapolydactyly, which is the presence of an extra appendage on all four limbs, is much more rarely encountered. Although pre-axial polydactyly is very common in White and Asian populations, post-axial polydactyly (PAP) is more common among the African American population [1]. Central polydactyly is the rarest of all types of polydactyly [1]. Many phenotypic variations exist in different subsets of populations based on race, ethnicity, and region. Hence, considerable genotypic variation is also expected. There are more than 20 different genes identified, out of which the genes involved in the anterior-to-posterior growth pattern are the most commonly involved (Shh-Gli pathway) [2-4]. This case report describes a 15-month-old female child who presented with findings of six digits on all four limbs and deviation of the left angle of mouth since birth with an echocardiography showing a large atrial septal defect measuring 7 mm in size, with a left-to-right shunt. Some of the most common syndromic associations seen along with polydactyly are Bardet-Biedl syndrome, Ellis-Van Creveld syndrome, Nager's syndrome, McKusick-Kaufman syndrome, and Weyer's syndrome. However, no known syndrome with a triad of tetrapolydactyly, congenital facial nerve palsy, and atrial septal defect is known to date. The closest syndromes from above that fit this triad are Ellis-Van Creveld syndrome, McKusick-Kaufman syndrome, and Holt-Oram syndrome, each of which shows polydactyly and congenital heart defects [5-7]. This article attempts to describe polydactyly in association with the most plausible syndromes pertaining to this particular case.

## Case presentation

**Figure 1:**
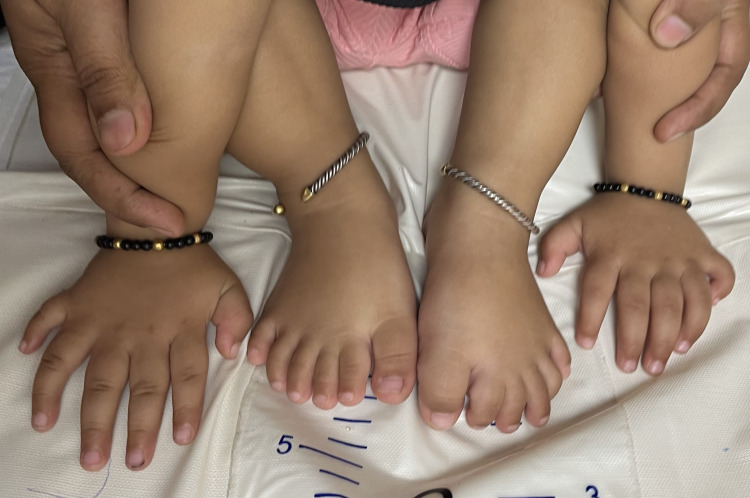
All four limbs showing central polydactyly (tetrapolydactyly)

**Figure 2:**
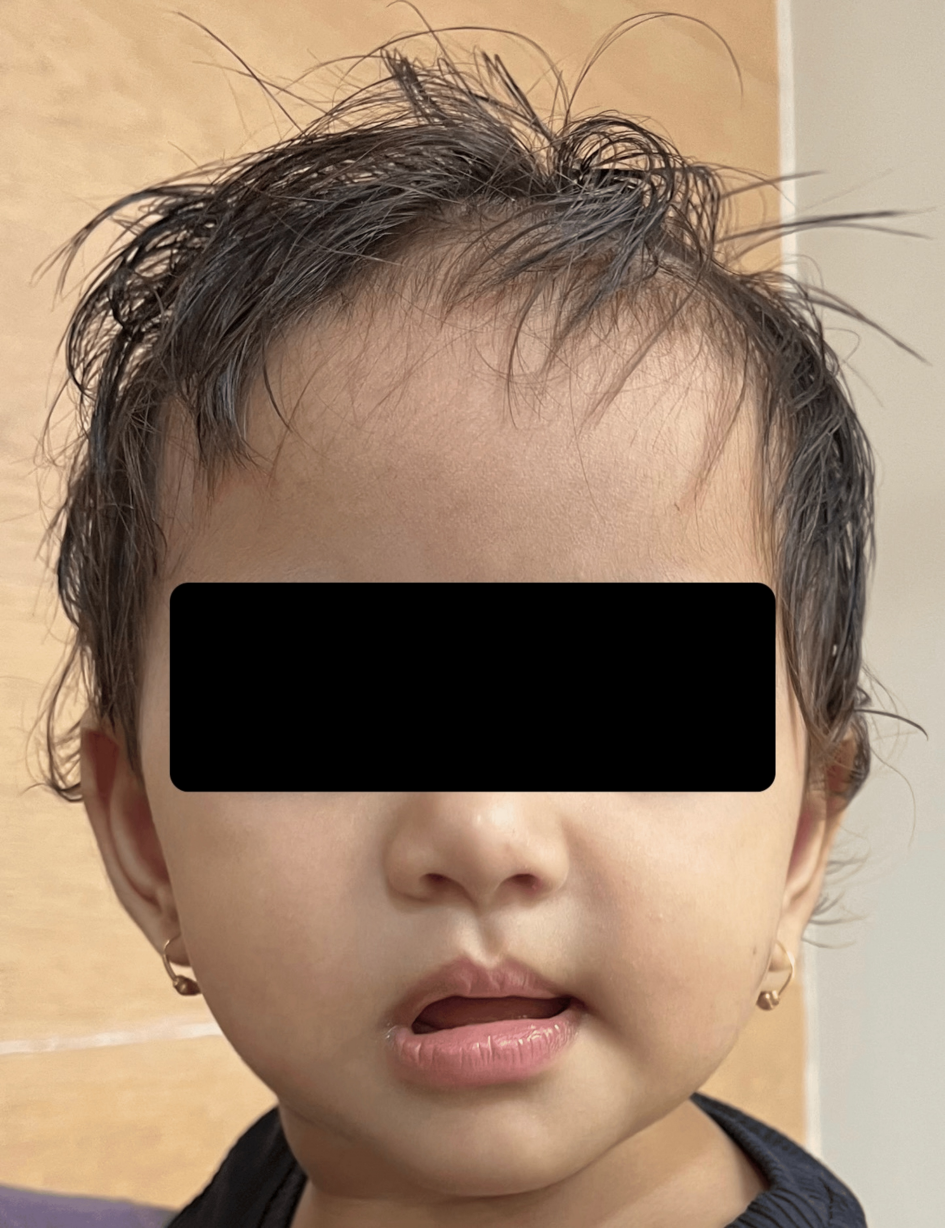
Deviation of the angle of the mouth toward the left side (present since birth)

A 15-month-old female child presented with findings of six digits on all four limbs (Figure 1) and deviation of the left angle of the mouth since birth (Figure 2). She was otherwise unremarkable on examination with normal neurodevelopmental milestones and growth centiles. She was born out of a non-consanguineous marriage and was the third by birth order, her mother having suffered a spontaneous abortion at two months of gestation before her birth, which was not investigated. At birth of the present female child, amniotic fluid was meconium stained, but she did not require resuscitation at birth and was admitted to the neonatal intensive care unit (NICU) for two days for the treatment of neonatal hyperbilirubinemia, which developed at 72 hours of life. In the NICU, she was evaluated with an echocardiography of the heart along with an ultrasound of her brain, abdomen, and pelvis to diagnose any associated birth defects with tetrapolydactyly. Her brain ultrasound and ultrasound of the abdomen and pelvis were normal. An echocardiography of her heart showed a large atrial septal defect measuring 7 mm, with a left-to-right shunt. There were no significant hemodynamic changes or remodeling of the heart at the time. She was advised annual echocardiography follow-up. She has yet to get her first follow-up echocardiography. She had no history suggesting a failing heart such as feeding difficulties, fatigue, recurrent respiratory infections, or growth failure. On auscultation of the pulmonary area of the chest, she had a grade 2 ejection systolic murmur. This was not associated with a thrill, distended neck veins, respiratory difficulty, edema, pallor, or cyanosis. She was neurologically and developmentally normal with a normal tone, power, and reflexes in all four limbs. All cranial nerves, except the left facial nerve, were normal on clinical examination. She had normal audiovisual activity, ocular movements, and pupillary reflexes. Her taste sensations and ability to swallow were intact. She had a normal gag reflex and could chew food properly for her age. There was no pooling or drooling of saliva from the left (affected) angle of the mouth. She could close both her eyes shut completely. Hence, there was an isolated upper motor neuron facial nerve palsy present. There was no finding of polydactyly, congenital heart disease (CHDs), or facial nerve palsy in her sibling, parents, or extended family. The mother had no history of ingestion of any kind of drugs other than calcium and iron supplements during the course of pregnancy.

## Discussion

**Figure 3:**
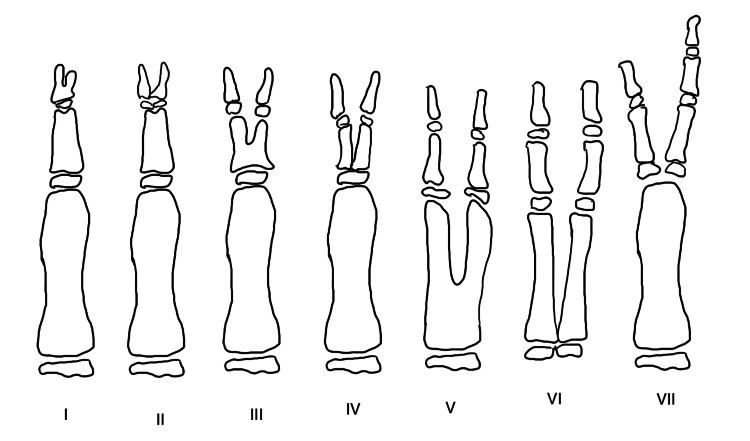
Wassel's classification of seven types of pre-axial polydactyly seen in the thumb Types I-VII of pre-axial polydactyly described by Wassel: type I, bifid distal phalanx; type II, complete duplication of the distal phalanx; type III, bifid proximal phalanx and a duplicated distal phalanx; type IV, complete duplication of the proximal and distal phalanges; type V, bifid first metacarpal with complete duplication of the proximal and distal phalanges; and type VI, complete duplication of the entire thumb ray (i.e., metacarpal and proximal and distal phalanges). Image courtesy: Janhavi V. Thorat

**Figure 4:**
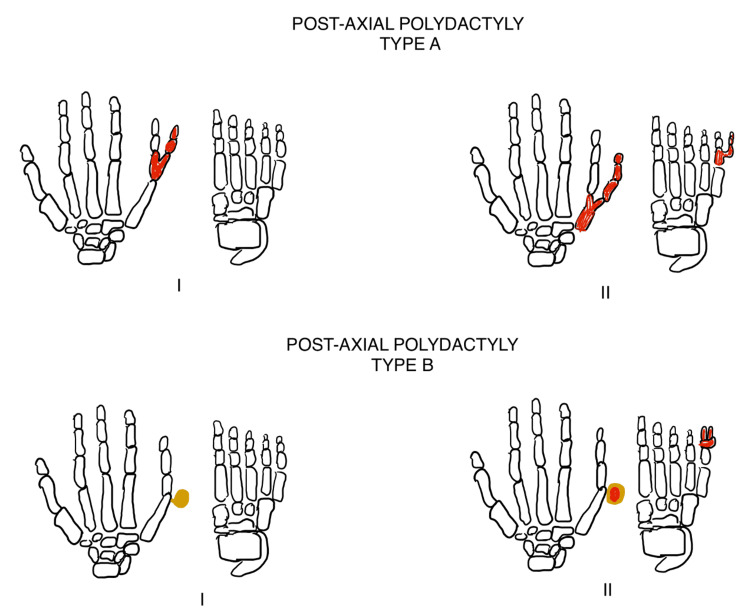
Temtamy and McKusick classification of post-axial polydactyly of the upper limb in conjunction with the type of polydactyly present in the lower limb Temtamy and McKusick classification of post-axial polydactyly of the upper limb in conjunction with the type of polydactyly present in the lower limb: type (A)(I) shows no abnormality in the metacarpals on the ulnar side of the hand with a bifid fifth proximal phalange of the hand and a normal foot, while type (A)(II) shows a bifid fifth metacarpal with a distal digit emerging post-axially from the bifid fifth metacarpal along with a bifid proximal phalange of the foot with a distal digit emerging from the said phalange of the foot, and type (B)(I) shows a pedunculated postminimus on the ulnar side of the hand with a normal foot, while type (B)(II) shows a pedunculated postminimus on the ulnar side of the hand along with a pedunculated postminimus on the fibular side of the foot [2]. Image courtesy: Janhavi V. Thorat

**Figure 5:**
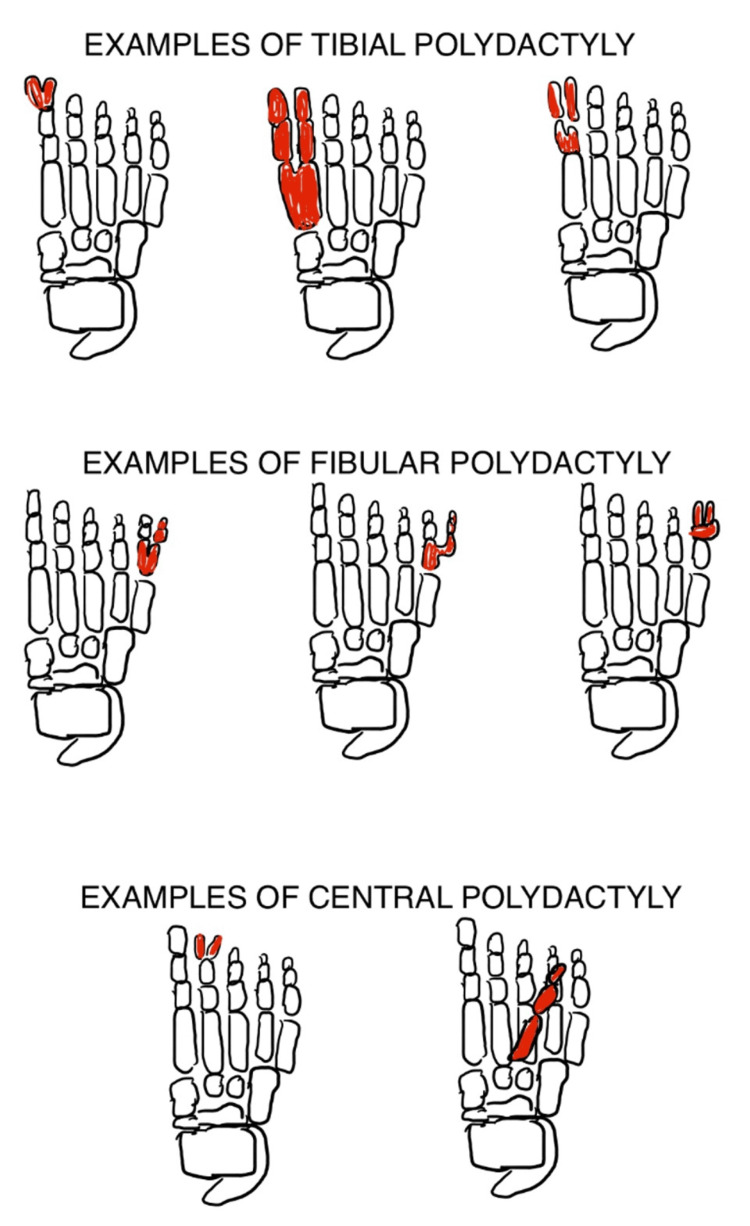
Examples of tibial polydactyly (affection on the side of the great toe), fibular polydactyly (affection on the side of the fifth toe), and central polydactyly (affection of any of the central three toes) Image courtesy: Janhavi V. Thorat

Polydactyly is the presence of an extra digit on the hand or the foot. It is an extremely common congenital anomaly encountered in children. It may be an isolated finding or may be found in conjunction with other congenital anomalies and syndromes [1]. Many classifications of polydactyly have been proposed. In the hand, it may occur as radial polydactyly (pre-axial polydactyly) or ulnar polydactyly (post-axial polydactyly) [1]. Depending upon the side of occurrence, it may be medial, that is, toward the little finger (called ulnar polydactyly) or lateral, that is, toward the thumb (called radial polydactyly). Radial polydactyly was classified further into seven different types by Wassel (Figure 3) [1], while Temtamy and McKusick classified ulnar polydactyly into two subtypes, A and B, in conjunction with the type of polydactyly present in the lower limb (Figure 4) [1,2]. On the feet, the extra digit can either be present on the side of the great toe (called tibial polydactyly) or on the side of the little toe (called fibular polydactyly) (Figure 5) [1,2]. In both the upper and the lower limbs, affection of any of the central three digits is called central polydactyly. Central polydactyly is the rarest of all types of polydactyly [1,2].

Studies place the incidence of polydactyly to be around 2.3 per 1,000 White males and 0.6 per 1,000 White females. This incidence jumps by about 10 times in African American males and 22 times in African American females [1]. Pre-axial polydactyly is more common in White and Asian populations with an incidence placed between 0.08 and 1.4 per 1,000 births [1]. However, post-axial polydactyly is more common among the two and seen more in the African American population as compared to other ethnicities [2,3]. Central tetrapolydactyly is a much rarer entity, and only a handful of case reports exist with no official incidence reported yet. Polydactyly presents with many phenotypic variations in different population groups based on race, ethnicity, and region. Hence, considerable genotypic variation exists. There are more than 20 different genes identified in association with polydactyly, out of which the genes involved in anterior-to-posterior growth pattern are the most commonly involved (Shh-Gli pathway). Some of the genes involved in the Shh-Gli pathway are *Shh*, *Gli-3*, *Smo*, and *Ptch-1* [4]. Other genes implicated are *TMEM237*, *TCTN3*,* NEK1*, *OFD1*,* DYNC2H1*, *EVC*, *MKS 1-10* (except *MKS-4*), *FGFR2*, *CD96*, *RAB23*, *MEGF8*,* EBP*, *GDF5*, *ICK*, *WNT7A*, and *GLI3* [4]. All these mutations cause various syndromes. Some of the most common syndromic associations are Bardet-Biedl syndrome, Ellis-Van Creveld syndrome, Nager's syndrome, and Weyer's syndrome. Syndromes can be further divided into ciliopathies and non-ciliopathies [4]. However, no known syndrome exists with a triad of tetrapolydactyly, congenital facial nerve palsy, and atrial septal defect, such as seen in this case. The closest syndromes fitting this constellation are Ellis-Van Creveld syndrome, McKusick-Kaufman syndrome, and Holt-Oram syndrome, each of which consists of polydactyly and congenital heart defects [5,6], but not facial nerve palsy. Congenital heart defects are one of the most common pediatric anomalies [8]. Ellis-Van Creveld syndrome presents with short stature, polydactyly, and congenital heart defects, which are seen in 50%-60% of those affected [5]. Orofacial-digital syndrome, also known as Mohr syndrome, shows anomalies of the digits, oral cavity, and face but does not involve the heart. McKusick-Kaufman syndrome has classical features of post-axial polydactyly (PAP), congenital heart disease (CHD), and hydrometrocolpos in females [6]. Features of all three syndromes have been described and compared in Table 1.

**Table 1:** Clinical features of common clinical syndromes associated with the features described in the patient

Syndrome	Clinical features in each syndrome	Limb anomaly	Congenital heart disease	Orofacial abnormality
Holt-Oram syndrome	-	Upper limb abnormality (most commonly absent radial bone)	Most commonly atrial septal defect or conduction abnormalities	Absent
Ellis-Van Creveld syndrome	-	Polydactyly (most commonly of the upper limb)	Most commonly atrial septal defect	High-arched palate, retrognathia, ectodermal dysplasia of teeth
Mohr syndrome	-	Polydactyly	Absent	Cleft lip, cleft palate
McKusick-Kaufman syndrome	-	Post-axial polydactyly	Atrial septal defect or ventricular septal defect	Absent

Atrial septal defects occur in about 25% of all children as a solitary finding or as part of a syndrome. They can be either of the following five types: patent foramen ovale (most common), ostium secundum defect, ostium primum defect, sinus venosus defect, or coronary sinus defect, which is the least common among all. Mechanisms of inheritance are many, such as mutations and transcription errors, to name a few [8]. The transcription factors involved in the septation of atria are *TBX5*, *GATA4*, and *Nkx2-5* [7]. Holt-Oram syndrome involves *TBX5* mutations and is associated with dysrhythmias and limb malformations [9]. Auscultation reveals an ejection systolic murmur over the pulmonary area with a fixed wide splitting of the second heart sound [10]. Defects less than 5 mm in size close spontaneously, while those greater than 10 mm require definite surgical closure [11].

Congenital facial nerve palsy can be attributed to many causes. In 50% of these cases, the etiology remains unknown [8]. Notable causes of facial nerve palsy are Bell's palsy, Moebius syndrome, and Goldenhar syndrome. Birth trauma is also one of the causes, and a birth weight of more than 3,500 g is a risk factor. Schwannomas and hemangiomas are other causes [12].

Only seven cases of tetrapolydactyly have been reported around the world. One case from 1995 describes the anomaly in a two-week-old male; however, the subtype has not been specified. There is no associated atrial septal defect or facial nerve palsy [13]. Another case is that of a five-year-old Iranian female who had retrognathia, high arched palate with a midline cleft lip (orofacial abnormalities), and tetrapolydactyly of the A4 subtype [14]. A case from Nigeria shows a newborn female with tetrapolydactyly with syndactyly of the A6 subtype [15]. Another case from Nigeria reported in 2023 describes a three-week-old male child with isolated tetrapolydactyly [16]. Only two cases have been reported in India, one from Kolkata [17] and the other from Bhubaneswar [18]. Even rarer is the association of tetrapolydactyly with congenital heart disease. Only one such report exists of a six-year-old Arab female who was described to have Caroli's disease along with the above two features [19], whereas no case in literature has described a case of central tetrapolydactyly with an atrial septal defect and congenital facial nerve palsy.

## Conclusions

Polydactyly, a very common congenital anomaly, should not be ignored in pediatric settings due to strong associations with various syndromes and anomalies of other systems. This patient was hence evaluated at birth for associated anomalies. It is important to diagnose associated features such as congenital heart diseases, genitourinary abnormalities, and orofacial abnormalities to facilitate timely surgical correction and help improve the quality of life of those affected. At-birth screening with echocardiography of the heart helped in the early diagnosis of atrial septal defect in this case. The patient's parents did not wish for surgical correction of tetrapolydactyly, and the harmless anomaly was hence allowed to persist. Congenital facial nerve palsy is currently being treated with physiotherapy. Annual follow-up with an echocardiography was advised, which is essential to prevent further complications with timely medical and surgical intervention. This triad of features remains to be described in any patient to date. This is the first such case reported from a tertiary care hospital with the aforementioned findings. It remains to be determined whether these are simply three unrelated features or the discovery of a new syndrome.
